# Expectation modulates the preferential processing of task-irrelevant fear in the attentional blink: evidence from event-related potentials

**DOI:** 10.1186/s12993-022-00203-6

**Published:** 2022-12-14

**Authors:** Meng Sun, Chenyang Shang, Xi Jia, Fang Liu, Lixia Cui, Ping Wei, Qin Zhang

**Affiliations:** 1 Learning and Cognition Key Laboratory of Beijing, School of Psychology, Capital Normal University, No.105, North Road of Western 3Rd-Ring, Beijing, 100048 China; 2 The School of Mental Health and Psychological Sciences, Anhui Medical University, Hefei, 230032 Anhui China; 3 Institute of Brain Science and Department of Psychology, School of Education, Hangzhou Normal University, Hangzhou, 311121 China; 4 Key Research Base of Humanities and Social Sciences of the Ministry of Education, Academy of Psychology and Behavior, Tianjin Normal University, Tianjin, 300387 China; 5 Faculty of Psychology, Tianjin Normal University, Tianjin, 300387 China

**Keywords:** Expectation, Fear, Attentional blink, Event-related potentials

## Abstract

**Background:**

Reporting the second of the two targets is impaired when it occurs 200–500 ms after the first, the phenomenon in the study of consciousness is the attentional blink (AB). In the AB task, both the emotional salience and the expectation of the second target increase the likelihood of that target being consciously reported. Yet, little is known about how expectations modulate the prioritized processing of affective stimuli. We examined the role of expecting fearful expression when processing fear in an AB task. Participants were presented with an AB task where the 2nd target (T2) is either a fearful face or a neutral face, and had to report the target's gender. The frequency of fearful to neutral faces on a given block was manipulated, such that participants could either expect more or less fearful faces.

**Results:**

In the Experiment 1, we found that fearful faces were more likely to be recognized than neutral faces during the blink period (lag3) when participants were not expecting a fearful face (low fear-expectation); however, high fear-expectation increased the discrimination of fearful T2 than neutral T2 outside the blink period (lag8). In the Experiment 2, we assessed ERP brain activity in response to perceived T2 during the blink period. The results revealed that fearful faces elicited larger P300 amplitudes compared to neutral faces, but only in the low fear-expectation condition, suggesting that expecting a fearful expression can suppress the processing of task-irrelevant facial expression and unexpected fearful expression can break through this suppression. Fearful T2 elicited larger vertex positive potential (VPP) amplitudes than neutral T2, and this affective effect was independent of fear-expectation. Since no effect of expectation was found on the VPP amplitude while P300 exhibited significant interaction between expectation and expression, this suggests that expectations modulate emotional processing at a later stage, after the fearful face has been differentially processed.

**Conclusions:**

These results provided clear evidence for the contribution of the expectation to the prioritized processing of second affective stimuli in the AB.

## Introduction

Rapid and accurate identification of facial emotions is a very important skill during social interactions. It has been showed that fearful expression relative to neutral expression automatically attracts attention and is preferentially processed [2, 28, 51].This was illustrated in an attentional blink (AB) paradigm in which participants were instructed to detect two targets (T1 and T2) among a rapid serial visual presentation (RSVP) of distractors at a rate of about 10 Hz. In this task, the detection ability of the second target (T2) is impaired when two targets are separated by a short temporal interval between 200 and 500 ms, a phenomenon called the ‘attentional blink’ [5, 38]. Interestingly, some studies have reported that emotional T2 attenuated the AB [2, 29, 37]. For example, participants were more likely to detect T2 if it was a fearful face instead of a neutral face in the AB task [7, 28].

Some researchers suggested that the enhanced bottom-up neural activation of coding for emotional stimuli was involved in the preferential attentional processing of emotional information [1, 6, 29]. The amygdala is thought to be involved in the early detection of emotional stimuli and facilitates the perception of emotional information via a substantial projection to the visual region [2, 7]. Many event-related potential (ERP) studies have shown rapid enhancement of sensory-perceptual processing (about 200 ms after T2 onset) at posterior sensors when participants detected an emotional T2 rather than a neutral T2 in an AB task [18, 23]. Other researchers proposed that in addition to this bottom-up mechanism, the top-down mechanism of expectation-driven biases to emotional information may also be involved in the attenuation effect of emotional T2 on AB [27, 29]. Participants might form expectations for the frequency of emotional stimuli based on the enhanced detection of emotional T2 in the previous trials, even though the frequency of the two types of stimuli was the same. Such emotional expectation could modify the perceptual processing of emotional stimuli in two top-down ways via top-down projections from higher-level brain regions, such as the frontal cortex [27, 46]. The first is that the expectation could speed up the perceptual processing of emotional stimuli by increasing the activation of sensory representations of emotional stimuli [12, 19, 21]. The other mechanism is that when the forthcoming emotional information is task-irrelevant, expectation could reduce the sensitivity of the brain to emotional stimuli by selectively suppressing the perceptual representation of emotional information [17, 53, 54]. However, what role emotional expectation plays in the attenuation effect of emotional T2 on attentional blink is still largely unknown.

Several findings suggested that the explicit relevance of expression to the task was a prerequisite for prioritized processing of facial expression [14, 44]. Indeed, the enhanced perception of emotional T2 stimuli compared with neutral T2 stimuli has been confirmed to be robust in the explicit emotional AB task, in which participants were instructed to attend to emotional features of T2 [6, 23, 24, 37, 43, 55]. However, whether attentional resources could be preferentially allocated to emotional information is controversial in the implicit emotional AB task in which emotional features were task-irrelevant. On the one hand, task-irrelevant expressions were found to facilitate the recognition of face identity in an implicit emotional AB task [3, 7]. For instance, Engen et al. [14] showed that fearful T2 could decrease the AB compared with neutral T2 even when participants reported the gender of facial stimuli. On the other hand, task-irrelevant expressions didn’t affect the performance in other implicit emotional AB tasks [44, 47]. For example, Sun et al. [47] showed that the prioritized processing of fearful facial expression in the AB task could only be observed when the facial expression had to be reported, but not when the faces’ gender was target. We speculated that the inconsistency of these results in the implicit emotional tasks was likely due to the top-down regulation of emotional expectation on the prioritized emotional processing, which might be suppressed when the expectation of upcoming emotional stimuli was formed.

Here, we primarily focused on the mechanism of how expectation affected the emotional processing in the implicit emotional AB task, in which T2 was either a fearful face or a neutral face and participants were instructed to report the gender of T2. Crucially, the probability of fearful T2 was manipulated to form emotional expectation by using the block-by-block method, so that fearful targets (60%) occurred more often than neutral targets (20%) or T2-absent distractors (20%) in the high fear-expectation block while the probability of fearful and neutral faces was 20% and 60% respectively in the low fear-expectation block. In Experiment 1, participants were instructed to report the scene of T1 and then discriminate the gender of T2 at the end of each stream. T2 was presented at third position (lag3; Stimuli Onset Asynchrony, SOA = 201 ms) or eighth position (lag8; SOA = 536 ms) after T1. The effect of emotional expectation on the preferential processing of fear was tested by comparing the correct reports of fearful T2 and neutral T2, given the T1 was identified correctly in the same trial. In this task, facial expression is task-irrelevant information. As one of the central cognitive functions of the human brain, expectation is generated by learning from prior experiences to optimize future behavioral responses [45]. The expectation of forthcoming fearful face based on its probability might modulate the representation of task-relevant facial expression. In an implicit emotional task, Yang et al. [53] reported that fearful faces and neutral faces elicited similar ERP responses in the expected condition, whereas unexpected fearful faces elicited increased P200 amplitudes than neutral faces, indicating that emotional expectation could suppress the brain susceptibility to task-irrelevant facial expression. Thus, we hypothesized that emotional expectation might inhibit the preferential processing of task-irrelevant facial expression in the high fear-expectation condition, thereby reducing the attenuation effect of fear on AB, while unexpected fearful faces might break out the suppressive effect, and then appear greater attenuation effect of fear on AB in the low fear-expectation condition.

In Experiment 2, we used the ERP to track the neural representations of T2 during the short T1-T2 interval (lag3). We proposed that the modulation effect of expectation might be related to the later processing represented by the P300, a large-amplitude late positive ERP component that appears in the central-parietal region, peaking at 300–600 ms after stimulus presentation [10, 35]. Numerous researchers have proposed that the P300 variously implicates the encoding of stimulus salience and probability [10, 13, 36], decision-making [39, 49], and context updating [34]. And in the AB task, the P300 is considered as an index of successful consolidation for the detected T2 [20, 56]. Specifically, we hypothesized that fearful faces would elicit enhanced P300 amplitudes compared to neutral faces only in the low fear-expectation condition. By contrast, this later emotional effect might be decreased or disappeared in the high fear-expectation condition. Previous studies have shown better performance for emotional T2 was associated with early enhanced perceptual processing reflected by early ERP components such as the vertex positive potential (VPP), which represented a stimulus-driven automatic attentional processing [18, 23]. And some top-down factors have little influence on this early emotional effect [22, 40]. Hence, we hypothesized that expectation did not influence the early emotional effect reflected by VPP, but inhibited the later consolidation of facial expression reflected by P300.

## Methods

### Participants

Thirty-two students participated in Experiment 1 (18 females; age, 18–26 years, 22.7 ± 2.1 years). Thirty students participated in Experiment 2 (17 females; age, 19–27 years, 22.2 ± 2.3 years). All participants were right-handed and had normal or corrected-to-normal vision. Due to high false alarm rate (incorrect response to T2 absent trials, ≥ 50%), two participants were excluded from analyses in Experiment 1 and one participant was excluded from analyses in Experiment 2. Furthermore, two participants with less than sixteen remaining epochs in the high-probability fear condition (usually for neutral faces, which had less trials) were excluded from ERP analyses in Experiment 2. In the end, we included 30 participants for Experiment 1 (17 females; age: 18–26 years, 22.8 ± 2.1 years), and twenty-seven participants for Experiment 2 (15 females; age, 19–27 years, 22.2 ± 2.4 years). All participants signed an informed consent form before the experiment. This study was approved by the Capital Normal University Institutional Review Board.

### Procedure and stimuli

Stimuli were presented using the Presentation software (Neurobehavioral Systems Inc., https://www.neurobs.com/presentation) and displayed on a 19-inch CRT monitor (1280 × 1024; 60 Hz) on a black (RGB:0, 0, 0) background. The viewing angle of each photograph was 5.73 × 8.37°. The participants were seated in a quiet, dimly-lit room, 100 cm away from the screen, and then performed an adapted RSVP task, in which a rapid series of visual stimuli for every trial consisted of two targets and 18 scrammed faces as distractors. We selected 40 copyright-released scenic photographs (half of the photographs depicted indoor scenes and the other half depicted outdoor scenes) from the Internet as T1 stimuli. The indoor scenes were photographs of drawing rooms and kitchens while the outdoor scenes were photographs of buildings, with no people or animals.

T2 stimuli included 40 faces (20 fearful faces and 20 neutral faces) taken from the NimStim face database (http://www.macbrain.org/resources.htm) [48], which consisted of half male and half female. These T2 stimuli were used in another study and their valence, arousal and fearfulness have been described previously [47]. The distractor items included 24 scrambled faces that were created by another six neutral faces selected from the NimStim face database. For every neutral face, their facial features were divided into 12 × 8 squares and randomly rearranged to a scrambled face. All scrambled faces and T2 faces were cropped into an oval shape to exclude hair, ears, and neck information, then converted into greyscale with constant luminance using the Adobe Photoshop CS5. The full-color scenic T1 stimuli were also cropped into an oval shape to control the stimuli size. The saliency of T1 stimuli was to make sure the identification of T1 and kept consistent in the high and low expectation blocks.

### Experiment 1

**Fig. 1:**
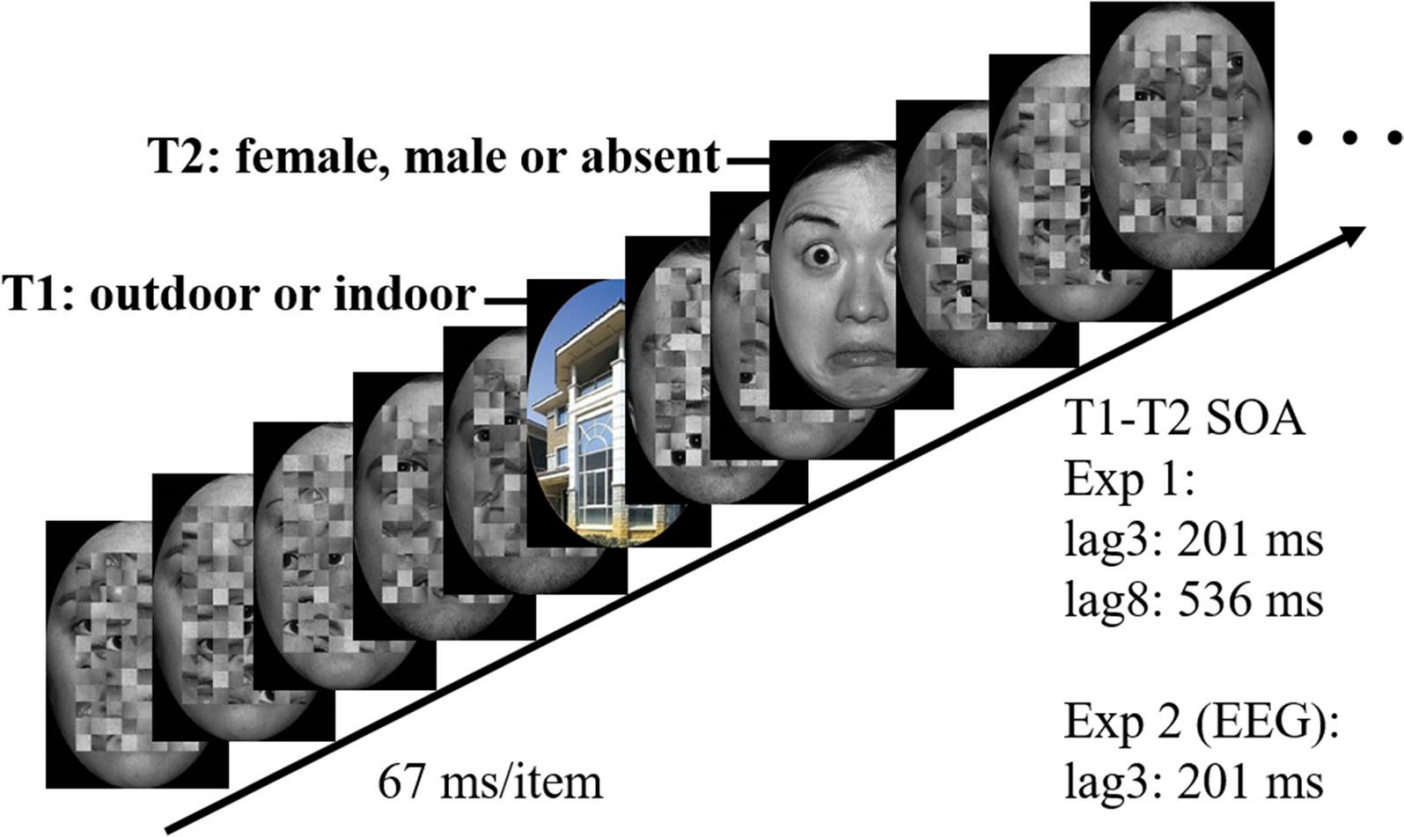
Task design of Experiment 1 and 2. Participants were instructed to judge the scene of the T1 (an outdoor or an indoor scene) and then judged the gender (female, male, or face absent) of the face in the T2

This was an implicit emotional expectation task and no verbal instruction about the high or low expectation of fear was given to participants. Participants were instructed to discriminate scene (T1: indoor scene or outdoor scene) and gender (T2: female or male) within a rapid series of scrambled faces. Each RSVP stream began with a 1 s presentation of a white fixation cross, followed by 20 sequential images that were presented for 67 ms each at the center of the screen (see Fig. 1). T1 was always presented at the sixth or eighth position of the RSVP. In most trials (80%), T1 was followed by a second target at lag3 (SOA = 201 ms), or lag8 (SOA = 536 ms). In the remaining trials (T2-absent trials, 20%), a scrambled face was presented at the time point where T2 supposed to be. At the end of each trial, participants were instructed to discriminate the scene of the first target by pressing button (the D key for “indoor” and the F key for “outdoor”), then judge the gender of the second target (the J key for “male face”, the K key for “female face”, the L key for “face absent”). The response timeout durations were 2 s and participants were instructed to focus on accuracy rather than response speed.

Experiment 1 included a practice block of 20 trials and four test blocks of 100 trials each. There were two high fear-expectation blocks and two low fear-expectation blocks. For high fear-expectation condition, 60% of trials were fearful T2 trials (60 trials per block), 20% were neutral T2 trials, and 20% were T2 absent trials. For low fear-expectation condition, 20% of trials were fearful T2 trials, 60% were neutral T2 trials, and 20% were T2 absent trials. The scene of T1 was balanced to the expression and gender of T2 stimuli. Participants practiced 20 trials to familiarize themselves with the procedure before the formal experiment. The order of the high fear-expectation condition and low fear-expectation condition was balanced across participants. Participants could take a short rest between blocks to avoid fatigue, and the whole experiment required about 40 min to complete.

### Experiment 2 (EEG)

The stimuli and procedure for Experiment 2 were identical to Experiment 1, except that the lag8 condition was absent in the Experiment 2. T2 was always presented at lag 3 (*i.e*., T2 and T1 were separated by 2 distractors) relative to T1. Again, Experiment 2 consisted of two high fear-expectation blocks and two low fear-expectation blocks (400 trials over four blocks). First, participants prepared for the EEG measurements and received brief instructions about the task. Then, participants performed four blocks of 100 trials each in the AB task.

EEG data were recorded by a NeuroScan Amplifier (Neuroscan SynAmps) with 64 electrodes embedded in an elastic cap using the extended 10–20 International System, along with one online reference electrode on the left mastoid and four electrodes measuring the vertical electrooculograms and the horizontal electrooculograms. The electroencephalogram was collected with a bandpass of 0.05–100 Hz during recording. The sampling rate was 500 Hz and the resistance of all electrodes was kept below 5 kΩ. After data acquisition, the offline EEG data were preprocessed with the EEGLAB toolbox (v14.1.1) for MATLAB-2015a [9]. First, data were re-referenced to the average of the left and right mastoids, high-pass filtered at 0.05 Hz, low-pass filtered at 30 Hz, and epoched from − 200 to 800 ms surrounding the onset of T2 stimuli. Baseline correction was applied using the average amplitude before T2 onset (− 200 to 0 ms). Second, we rejected some significant artifacts (i.e., large muscle activity produced by cough or swallow) not related to eye blinks with visual inspection. As a final step, the independent component analysis (ICA) was used to identify and remove components associated with eye blinks and muscle tension from the EEG data [4]. In this step, we used the EEGLAB’s default algorithm ‘runica’, which perform ICA decomposition of input data using the logistic infomax ICA algorithm with the natural gradient feature. Only trials with correct responses to both T1 and T2 targets were included for EEG analyses. The numbers of epochs retained for analyses (mean, median and range) for each condition of interest were as follows: high probability condition, fearful faces (Mean ± SD, 89.2 ± 20.7, Median: 91, Range: 38–114), neutral faces (Mean ± SD, 30.6 ± 5.6, Median: 30, Range: 19–40); low probability condition, fearful faces (Mean ± SD, 30.6 ± 6.7, Median: 32, Range: 16–39), neutral faces (Mean ± SD, 92.7 ± 16.3, Median: 99, Range: 56–113).

In Experiment 2, we mainly focused on the VPP and P300 components elicited by T2 stimuli. T2-locked average ERPs under different conditions were computed separately for each participant as the difference between T1-T2 trials and T2-absent trials (i.e. the average ERP of fearful faces condition subtracts the average ERP in T2-absent trials). The choices about electrode sites of VPP and P300 components was based on previous studies with a similar design to the present study [23, 47]. And the choices of time window for certain ERP components were based on the grand-averaged ERP activity of the present study. We calculated mean amplitude in the relevant time window symmetrically centered around the peak latency for each component, with a shorter time window length (40 ms) for the VPP components and a longer time window length (100 ms) for the P300 component. We calculated the VPP amplitudes at the electrode sites of FC3, FCz, FC4, C3, Cz, and C4 between 180 and 220 ms. The P300 amplitudes were measured with FC3, FCz, FC4, C3, Cz, C4, CP3, CPz, CP4, P3, Pz, and P4 from 470 to 570 ms.

### Statistical analysis

#### Experiment 1

First, T1 accuracy was analyzed by using a 2 × 2 × 2 repeated-measures analyses of variance (ANOVA) with three factors which are fear-expectation (high, low), expression (fearful, neutral), and lag (Lag3, Lag8). Then, we analyzed the percentage of correct T2 reports from trials in which T1 was accurately identified (T2|T1), consistent with the previous study [38]. First, we calculated T2 accuracy based on the correct reports of T2-presented trials and correct rejections of T2-absent trials. T2 accuracy was analyzed by using a 2 × 2 × 2 repeated-measures analyses of variance (ANOVA) with three factors which are fear-expectation (high, low), expression (fearful, neutral), and lag (Lag3, Lag8).

#### Experiment 2 (EEG)

The method of behavioral analyses for Experiment 2 were similar to those for Experiment 1. However, Experiment 2 did not conclude factor lag. T2 behavioral performance was analyzed by using a 2 × 2 repeated-measures ANOVA with the factors fear-expectation (high, low), and expression (fearful, neutral). All EEG analyses were based on trials where T1 and T2 were correctly identified. The two-way repeated measures ANOVAs on the mean amplitudes of the VPP and P300 components were performed with the fear-expectation (two levels: high and low), and expression (two levels: fearful and neutral), as within-subjects factors. P values were corrected by the Greenhouse–Geisser correction. Bayesian analyses were performed to quantify the evidences for the null hypothesis using JASP 0.10.2.0 (JASP Team, 2019, Amsterdam, The Netherlands), with default JASP Cauchy priors. We computed the Bayes Factor (BF) of each effect with BF_10_ denoting the evidence for the alternative hypothesis and BF_01_ denoting the evidence for the null hypothesis. We interpreted BF from 1 to 3 as weak evidence in favor of either hypothesis, values from 3 to 10 as moderate, and those above 10 as strong evidence in favor of either conclusion [16].

## Results

### Experiment1

First, the T1 performance was analyzed. The mean accuracy of T1 discrimination across all conditions was 92.7 ± 1.2% (M ± SE, the same below). A three-way ANOVA with factors of the fear-expectation (high, low), expression (fear, neutral) and lag (lag3, lag8) was conducted on T1 accuracy. The main effect of lag was significant [*F*(1, 29) = 4.95, *p* = 0.034, *η*2 p = 0.15, BF_10_ = 1.21], with higher T1 accuracy at the lag3 condition (93.4 ± 1.1%) than at the lag8 condition (92 ± 1.3%). Other main effects [fear-expectation: *F*(1, 29) = 0.79, *p* = 0.38, BF_01_ = 5.2; expression: *F*(1, 29) = 0.798, *p* = 0.38, BF_01_ = 4.99] and interactions [fear-expectation × expression: *F*(1, 29) = 2.02, *p* = 0.17, BF_01_ = 2.53; fear-expectation × lag: *F*(1, 29) = 0.59, *p* = 0.45, BF_01_ = 3.74; expression × lag: *F*(1, 29) = 0.32, *p* = 0.58, BF_01_ = 3.56; fear-expectation × expression × lag: *F*(1, 29) = 1.38, *p* = 0.25, BF_01_ = 1.97] were not significant.

**Fig. 2:**
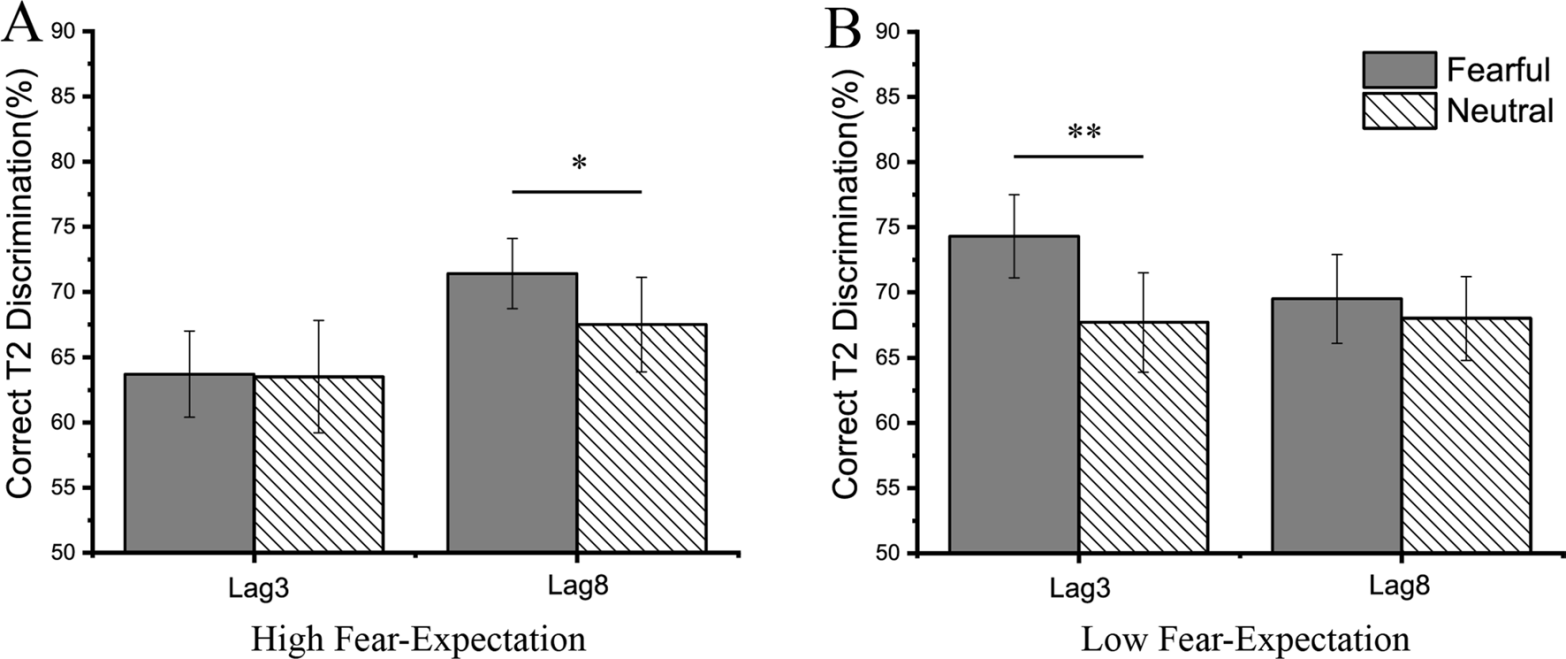
Behavioral results of Experiment 1. Mean percentage of the correct T2 identification for fearful and neutral faces in the high fear-expectation condition **A** and in the low fear-expectation condition **B**, depicted separately for the lag3 and lag8 condition

**Fig. 3:**
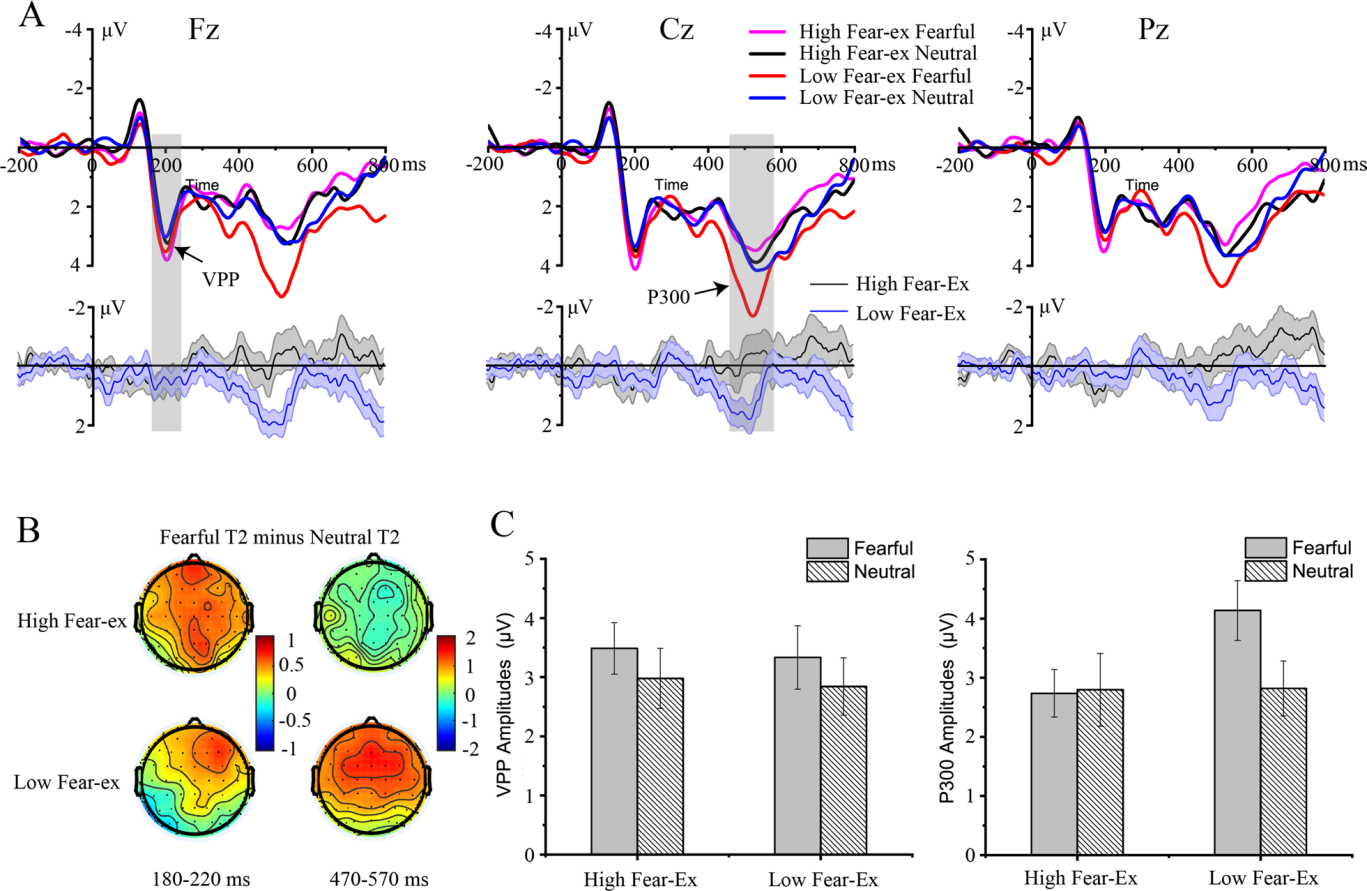
ERP data of the correct discrimination for the T1 and T2 in Experiment 2. Grand-average ERPs for fearful (High Fear-ex Fearful, magenta line) and neutral faces (High Fear-ex Neutral, black line) in the high fear-expectation condition, and fearful (Low Fear-ex Fearful, red line) and neutral faces (Low Fear-ex Neutral, blue line) in the low fear-expectation condition at the Fz, Cz, and Pz **A**; the difference waveforms and 95% confidence interval of ERPs generated by fearful faces minus neutral faces for the high fear-expectation condition (black line) and the low fear-expectation condition (blue line) at the Fz, Cz, and Pz **A**. The scalp topographies of difference waves between fearful faces and neutral faces in the high and low fear-expectation conditions at 180–220 ms and 470–570 ms **B**. The bar graphs showing the average amplitude of the four conditions (the fear-expectation × expression) for VPP and P300 **C**

The mean accuracy of T2 discrimination across all conditions was 68.1 ± 3.1%. An ANOVA with the fear-expectation (high, low), expression (fear, neutral) and lag (lag3, lag8) as factors on T2 discrimination was performed (Fig. 2). The main effect of fear-expectation was significant [*F*(1, 29) = 8.05, *p* = 0.008, *η*2 p = 0.22, BF_10_ = 3.22]. T2 performance was significantly better in the low fear-expectation condition (69.7 ± 3.1%) than that in the high fear-expectation condition (66.5 ± 3.2%). The main effect of expression was significant [*F*(1, 29) = 4.71, *p* = 0.04, *η*2 p = 0.14, BF_10_ = 1.96], with higher T2 accuracy for fearful T2 (69.6 ± 2.8%) than for neutral T2 (66.7 ± 3.5%). Besides, the interaction between the fear-expectation and lag was significant [*F*(1, 29) = 13.96, *p* = 0.001, *η*2 p = 0.33, BF_10_ = 16.8]. Simple effect analysis revealed that there was no significant difference in mean accuracy between high fear-expectation condition (69.3 ± 3.1%) and low fear-expectation condition (68.7 ± 3.2%) in the lag8 condition (*p* = 0.605), but the accuracy in high fear-expectation condition (63.7 ± 3.7%) was lower than that in the low fear-expectation condition (70.8 ± 3.3%) in the lag3 condition (*p* < 0.001). More importantly, the interaction of the fear-expectation, expression and lag was significant [*F*(1, 29) = 5.32, *p* = 0.03, *η*2 p = 0.16, BF_10_ = 0.56]. Further simple effect analysis revealed that in the high fear-expectation condition, there was a significant expression effect in the lag 8 condition only, with significantly higher accuracy of fearful faces than neutral faces [*F*(1, 29) = 4.92, *p* = 0.04, *η*2 p = 0.15, BF_10_ = 1.73; fear: 71.2 ± 2.7%; neutral: 67.5 ± 1.6%]. In addition, in the low fear-expectation condition, the expression effect was significant in the lag 3 condition only, with significantly higher accuracy of fearful faces than neutral faces [*F*(1, 29) = 7.4, *p* = 0.01, *η*2 p = 0.2, BF_10_ = 4.16; fear: 74.0 ± 3.3%; neutral: 67.6 ± 3.8%]. The main effect of lag [*F*(1, 29) = 0.78, *p* = 0.38, BF_01_ = 2.53] and other interactions [fear-expectation × expression: *F*(1, 29) = 1.29, *p* = 0.27, BF_01_ = 3.46; expression × lag: *F*(1, 29) = 0.24, *p* = 0.63, BF_01_ = 4.28] were not significant Fig. 3.

We split the sample to “blinkers” (who do show an AB) and “non-blinkers” (who do not show an AB) based on whether participants showed an AB effect in the neutral condition or not. AB magnitude was calculated as the percentage of decrement in T2 performance (given that T1 was accurately identified) relative to T1 performance at the lag3 condition according to the following formula: (T1_lag3_-T2|T1_lag3_)/T1_lag3_ × 100%. According to the criteria of blinkers and non-blinkers proposed by Martens and Valchev [25], six participants with an AB magnitude of 10% or less were classified as non-blinkers (mean = − 3.03%), another twenty-four participants as blinkers (mean = 36.12%). For blinkers, an ANOVA with the fear-expectation (high, low), expression (fear, neutral) and lag (lag3, lag8) as factors on T2 discrimination was performed. Still, we observed a similar pattern of results. The main effects of the fear-expectation [*F*(1, 23) = 11.93, *p* = 0.002, *η*2 p = 0.34, BF_10_ = 5.04] and the expression [*F*(1, 23) = 10.15, *p* = 0.004, *η*2 p = 0.31, BF_10_ = 19.93] were significant. The interaction between the fear-expectation and lag was significant, *F*(1, 23) = 15.69, *p* = 0.001, *η*2 p = 0.41, BF_01_ = 27.17. And the interaction of the fear-expectation, expression and lag was significant [*F*(1, 23) = 6.06, *p* = 0.022, *η*2 p = 0.21, BF_10_ = 1.19]. Further simple effect analysis revealed that in the high fear-expectation condition, there was a significant expression effect in the lag 8 condition only, with significantly higher accuracy of fearful faces than neutral faces (fear: 68.6 ± 3.1%; neutral: 63.3 ± 4.0%, *p* = 0.01]. In addition, in the low fear-expectation condition, the expression effect was significant in the lag 3 condition only, with significantly higher accuracy of fearful faces than neutral faces (fear: 71.7 ± 3.7%; neutral: 62.7 ± 4.1%, *p* = 0.001]. The main effect of lag [*F*(1, 23) = 1.28, *p* = 0.27, BF_01_ = 1.19] and other interactions [fear-expectation × expression: *F*(1, 23) = 1.22, *p* = 0.28, BF_01_ = 3.89; expression × lag: *F*(1, 23) = 0.85, *p* = 0.37, BF_01_ = 4.32] were not significant.

### Experiment 2

#### Behavioral results

The mean accuracy of T1 across all conditions was 96.7 ± 0.6%. A three-way ANOVA with factors of the fear-expectation (high, low) and expression (fear, neutral) was conducted on T1 accuracy. The main effects of the fear-expectation [*F*(1, 26) = 1.64, *p* = 0.21, BF_01_ = 1.19] and the expression [*F*(1, 26) = 2.31, *p* = 0.14, BF_01_ = 3.27], as well as the interaction between the expression and the fear-expectation [*F*(1, 26) = 0.01, *p* = 0.99, BF_01_ = 3.69] were not significant.

The mean accuracy of T2 across all conditions was 78.9 ± 2.7%. A two-way ANOVA with factors of the fear-expectation (high, low) and expression (fear, neutral) performed on T2 discrimination did not reveal significant main effects of the fear-expectation [*F*(1, 26) = 2.99, *p* = 0.096, BF_01_ = 1.02] and the expression [*F*(1, 26) = 0.37, *p* = 0.546, BF_01_ = 4.06]. The interaction effect between the fear-expectation and the expression was also not significant [*F*(1, 26) = 0.64, *p* = 0.43, BF_01_ = 3.14].

According the AB magnitude, seven participants with an AB magnitude of 10% or less were classified as non-blinkers (mean = 4.94%) and another twenty participants as blinkers (mean = 22.89%). Again, a two-way ANOVA with factors of the fear-expectation (high, low) and the expression (fear, neutral) was performed on T2 discrimination for blinkers did not reveal significant main effects of the fear-expectation [*F*(1, 19) = 3.09, *p* = 0.095, BF_01_ = 0.92] and the expression [*F*(1, 19) = 0.26, *p* = 0.618, BF_01_ = 3.49], as well as the interaction effect [*F*(1, 19) = 0.32, *p* = 0.578, BF_01_ = 3.53].

#### ERP results

##### VPP (180–220 ms)

There was a significant main effect of the expression [*F*(1, 26) = 4.4, *p* = 0.046, *η*2 p = 0.15, BF_10_ = 0.66], with larger VPP amplitudes elicited by fearful faces (3.409 μV) than neutral faces (2.911 μV) (Fig. 3). No further main effect of the fear-expectation [*F*(1, 26) = 0.109, *p* = 0.744, BF_01_ = 4.593] or interaction was significant [*F*(1, 26) = 0.002, *p* = 0.968, BF_01_ = 3.599].

##### P300 (470–570 ms)

There was no significant main effect of the fear-expectation [*F*(1, 26) = 2.798, *p* = 0.106, BF_01_ = 0.7] or the expression [*F*(1, 26) = 1.985, *p* = 0.171, BF_01_ = 2.16]. However, the interaction between the fear-expectation and the expression was significant [*F*(1, 26) = 8.147, *p* = 0.008, *η*2 p = 0.24, BF_10_ = 0.972] (Fig. 3). A simple effect analysis revealed that there was no significant difference between the fearful faces (2.798 μV) and neutral faces (2.948 μV) in the high fear-expectation condition (*p* = 0.758, BF_01_ = 3.594). However, fearful faces (4.227 μV) elicited larger P300 amplitudes than neutral faces (3.076 μV) in the low fear-expectation condition (*p* = 0.003, BF_10_ = 11.936).

We analysis these ERP results only for blinkers. For the VPP, the main effects of the fear-expectation [*F*(1, 19) = 0.004, *p* = 0.951, BF_01_ = 4.35] and the expression [*F*(1, 19) = 4.01, *p* = 0.06, BF_01_ = 1.64] were not significant; and the interaction between the fear-expectation and the expression was also not significant [*F*(1, 19) = 0.156, *p* = 0.697, BF_01_ = 3.11]. Again, we observed similar results of P300 in blinkers. The interaction between the fear-expectation and the expression was significant [*F*(1, 19) = 10.219, *p* = 0.005, *η*2 p = 0.35, BF_10_ = 1.51]. A simple effect analysis revealed that there was no significant difference between the fearful faces (2.91 μV) and neutral faces (3.28 μV) in the high fear-expectation condition (*p* = 0.549, BF_01_ = 2.8). However, fearful faces (4.96 μV) elicited larger P300 amplitudes than neutral faces (3.58 μV) in the low fear-expectation condition (*p* = 0.006, BF_10_ = 7.2). The main effect of the fear-expectation was significant [*F*(1, 19) = 5.023, *p* = 0.037, BF_10_ = 4.31], while the main effect of the expression were not significant [*F*(1, 19) = 1.239, *p* = 0.28, BF_01_ = 2.42].

## Discussion

In the present study, we manipulated the probability of fearful T2 in an implicit emotional AB task to test how the expectation of fearful faces influenced the effect of fear on AB. First, the experiment 1 revealed that fearful expression facilitated the discrimination of gender within the blink period only in the low fear-expectation condition, but not in the high fear-expectation condition, suggesting that unexpected fearful expression attenuated AB in the implicit emotional AB task. Secondly, the modulation effect of emotional expectation on emotional processing during the AB task could be observed on the P300 component, with larger P300 amplitudes elicited by fearful faces than neutral faces in the low fear-expectation condition, but not in the high fear-expectation condition. Furthermore, fearful faces elicited early enhancement on VPP amplitudes than neutral faces, which was independent of the fear-expectation. However, the behavioral effects of expectation on expression were not replicated in the experiment 2. Taken together, these results suggested that unexpected fearful faces might attenuate AB and get the preferential processing during the implicit emotional AB task.

In the implicit emotional task, whether emotional T2 attenuated AB depended on the expectation for task-irrelevant emotional information. Previous studies have shown that emotional T2 could attenuate the AB in the explicit emotion task [14, 23, 24, 37, 43]. Extending these previous findings, the present study found that advantage for fearful versus neutral faces only in the low fear-expectation condition for lag 3 (i.e., within the blink period) in the implicit emotion task. The most well-supported theories of the AB [5, 32, 52] suggest that the AB reflects the suppression of attentional engagement during the blink period. Thus, the advantage of unexpected fearful faces in the lag3 condition suggested that unexpected threatening stimuli could break through the suppression that occurs during the blink. This may be an evolutionary advantage, that is, the brain may be more sensitive to unexpected threatening stimuli that could devote more attentional engagement to process these stimuli [15, 33]. In addition, the same advantage for fearful versus neutral faces was only in the lag8 condition (i.e., outside the blink period) when fearful faces were expected, suggesting that fearful faces could capture attention again when the suppression of attentional engagement was relieved in the lag8 condition. In the AB task, the available attentional resources of T2 depended on its interval from T1 [5, 11]. Thus, the factor of T1-T2 interval actually isolated the effects of expectation and attention. The different pattern of emotional expectation on emotional processing in two T1-T2 interval condition indicated that expectation and attention might interact to produce this effect. We speculated that in the emotional implicit AB task, participants might establish an attentional template based on high anticipation of emotional stimuli to selectively inhibit the processing of task-irrelevant emotional information, but this attentional template might only play a role when attentional resources were relatively insufficient (lag3). Thus, the advantage of expected fearful faces in the lag3 condition was disappeared because the processing of expression was suppressed based on the attentional template.

When the probability of emotional stimuli was low or the attentional resources were sufficient (lag8), the suppression of task-irrelevant emotional information based on attentional template would be broken, and the facial expression would get the preferential processing again. However, it should be noted that there seems to be a small lag effect in the high fear-expectation for both the neutral and fearful expression conditions, although the paradigm that we used was robust enough to produce an AB effect [29, 47]. To excluded the effect of an individual difference in the AB effect, we split the sample to “blinkers” and “non-blinkers” based on previous reported method [25]. Our additional analyses excluded these non-blinkers and found similar main effects and interaction effects as original analyses, suggesting that the effect of expectation on expression cannot be explained by individual differences in the AB effect.

The neural mechanism underlying the expectation effect was measured with EEG in Experiment 2. Consistent with a previous study [47], we found that fearful faces elicited increased VPP amplitudes than neutral faces, and this emotional effect was independent of expectation. Previous studies using explicit or implicit emotion tasks have found this early effect, which was considered to reflect the rapid detection of fear by the amygdala [2, 23, 31]. Here, since facial expression was a task-independent feature in this study, we concluded that this early emotional effect was related to the bottom-up automatic attentional capture of facial expression. However, the significant main effect of expression on the VPP was not supported by Bayes factors in the present study (BF_10_ = 0.66), whereas this effect was supported strongly by Bayes factors in our previous study with a BF_10_ of 24.22 [47]. Reporting both p-values and BF might result in effects falling into different categories of statistical decision-making, especially if statistical power is low. Hence, more studies are required to focus on this contradiction in the future. More importantly, our findings revealed that expectation modulated the later working memory consolidation of emotional stimuli, reflecting by the P300 component. Specifically, we found that fearful T2 triggered a larger P300 amplitudes than neutral T2 in the low fear-expectation condition. By contrast, P300 amplitudes were similar for fearful and neutral T2 in the high fear-expectation condition. These results were consistent with one study reported by Yang et al. [53], who proposed that emotional expectation could decrease the brain sensitivity to fearful stimuli in an implicit emotional task. In an AB task, the detected T2 evoked larger P300 amplitude compared to the missed T2, indicating that the target was encoded into working memory [8, 20, 26, 56]. Thus, our findings suggested that in the low fear-expectation condition, unexpected fearful faces captured more attentional resources and were encoded into working memory. In Experiment 2, we observed that expectation suppressed the later processing of facial expression reflecting by P300, but did not affect the early emotional effect. These results are consistent with a recent view that expectation has little influence on early sensory responses and primarily influences later elaborate stages of information processing [40]. However, these explanations could only be limited in the condition that the attentional resources were insufficient, because Experiment 2 only recorded neural activities during the short T1-T2 interval. Future research could also focus on the neural mechanisms of expectation on emotional processing when attentional resources were relatively sufficient.

For the modulation effect of expectation on the later processing of emotional stimuli, an alternative explanation was that the increased P300 amplitudes for unexpected fearful faces compared with neutral faces might merely reflect the probability of stimuli. The P300 is sensitive to stimuli probability, with unexpected or deviant stimuli eliciting a larger P300 than high probability stimuli [34]. Besides, several emotional studies have showed that negative relative to neutral facial expressions usually induce larger amplitudes of later positive potential (LPP), which has a similar distribution and time course as the P300, reflecting the sustained engagement and elaborate processing for emotional stimuli [13, 41]. Thus, it seems possible that the larger LPP for negative faces and the larger P300 for neutral faces (due to their lower probability) in the high-fear-expectation condition might have canceled out, while both effects would go in the same direction (i.e., larger LPP and larger P3 for fearful faces) in the low-fear-expectation condition. If this alternative explanation was right, we would also observe the probability effect of neutral faces, with larger P300 for neutral faces in the high fear-expectation condition than that in the low fear-expectation condition. However, this speculation was not supported by our ERP data that low-probability neutral faces did not elicit larger P300 amplitudes. In addition, our previous study that included a condition with equal probabilities for fearful and neutral faces in the implicit emotional AB task might help to quantify potential influences of fearful expressions on the P300 [47]. In that study, we found that fearful faces elicited similar P300 as neutral faces when participants reported the target’s gender. This result suggested that facial expression was not processed in the later stage. Hence, the similar P300 of fearful and neutral faces in the high fear-expectation condition was not due to the emotional salience of fearful faces and the lower probability of neutral faces. These evidences indicated that the effect of expectation on the P300 in the implicit emotional AB task did not merely reflect the stimulus probability.

In the present study, the probability of fearful T2 was manipulated to investigate the effect of expectation on the prioritized emotional processing during the implicit emotional AB task. The data indicated that emotional expectation modulated the prioritized processing of fear in the later working memory consolidation stage. We mentioned earlier that emotional expectation may modify the perception of emotional stimuli through two top-down mechanisms, enhancing or inhibiting the perceptual representation of emotional information [46]. Since facial expression was task-irrelevant information in the present study, the later top-down mechanism of inhibition was considered to involve in the effect of emotional expectation on emotional processing, by selectively suppressing the later neural representation of facial expression. However, the present study did not find the evidence that emotional expectation could modulate the early emotional effect, which reflected a bottom-up automatic processing driven by the emotional salience of facial expression [23, 47]. The bottom-up process of emotional stimuli may only facilitate the early detection of the target, and the top-down process driven by emotional expectation may trade off the facilitation effect of expression on target discrimination. In general, two main conclusions can be drawn from the two experiments. First, expectation traded off the attenuation effect of fear on AB in the implicit emotional task. By contrast, unexpected fearful faces could attenuate AB compared with neutral faces in the low fear-expectation condition even when facial expression was task-irrelevant. Second, emotional expectation primarily influences the later processing of facial expression reflecting by P300, but has little influence on the early emotional effect. These results suggest an important role of top-down expectation in the preferential processing of fear under limited attentional resources, that is, expectation for task-irrelevant facial expression and the emotional salience of stimuli can interact to affect the perception of emotional stimuli.

Our findings provide new evidence supporting the hypothesis that task-irrelevant emotional expectation may modulate the prioritized processing of fear, via suppressing the neural representation of facial expression in the later working memory consolidation stage. However, one limitation should be noted that the behavioral results of Experiment 2 did not replicate the expectation effect as Experiment 1, indicating that the ERP results during Experiment 2 might not be driven by the behavioral effects as Experiment 1. We have made a specific hypothesis that fearful faces might elicit enhanced later brain activity than neutral faces only in the low fear-expectation condition, but not in the high fear-expectation condition. The ERP results of Experiment 2 were consistent with our hypothesis, but the lack of a behavioral effect of fear in the low fear-expectation condition prevented us from drawing strong conclusions. Thus, further research is needed to verify this link that the ERP effects we observed in Experiment 2 are critical for behavior in the AB task of Experiment 1. In addition, we speculated that the lack of behavioral effects might be related to the increased temporal expectation in Experiment 2. Previous studies have indicated that temporal expectation also attenuated AB when the position of target was predictable [42, 50]. In the present study, two T1-T2 lags were used in Experiment 1, whereas only one lag3 was used in Experiment 2 in which the position of T2 was predictable. Indeed, we observed higher T2 accuracy in Experiment 2 (78.9 ± 2.7%) than that in Experiment 1 (67.3 ± 3.4%) in the lag3 condition, suggesting that the implicit temporal expectation induced by fixed T1-T2 interval facilitated the detection of T2 in Experiment 2. Therefore, the facilitation effect of temporal expectation might potentially mask the attentional capture effect of unexpected fearful faces. But future studies are still needed to look at this issue.

Another limitation of this study is that only fearful and neutral faces were compared. In previous studies, fear and anger expressions signal threats in the environment, and these negative expressions can quickly capture the individual’s attention and attenuate AB [3, 24, 28]. Some researchers believe that the effect of emotion on AB is mainly related to the arousal of stimulus, and positive expression can also attenuate AB [30, 37]. Therefore, we speculated that the interaction of expectation and fear in AB found in this study could be extended to expressions of anger, as well as positive expression with higher arousal. Future research should continue to explore this issue.

## Data Availability

The data are currently not publicly available due to participant privacy.
